# Italian Guidelines for the Management of Sporadic Primary **Hyperparathyroidism**

**DOI:** 10.2174/0118715303260423231122111705

**Published:** 2024-04-19

**Authors:** Fabio Vescini, Giorgio Borretta, Iacopo Chiodini, Marco Boniardi, Marina Carotti, Elena Castellano, Cristiana Cipriani, Cristina Eller-Vainicher, Sandro Giannini, Maurizio Iacobone, Antonio Stefano Salcuni, Federica Saponaro, Stefano Spiezia, Annibale Versari, Guido Zavatta, Zuzana Mitrova, Rosella Saulle, Simona Vecchi, Debora Antonini, Michele Basile, Alexia Giovanazzi, Agostino Paoletta, Enrico Papini, Agnese Persichetti, Irene Samperi, Alessandro Scoppola, Roberto Novizio, Pietro Giorgio Calò, Filomena Cetani, Luisella Cianferotti, Sabrina Corbetta, Maria Luisa De Rimini, Alberto Falchetti, Giovanni Iannetti, Stefano Laureti, Celestino Pio Lombardi, Bruno Madeo, Claudio Marcocci, Sandro Mazzaferro, Vittorio Miele, Salvatore Minisola, Andrea Palermo, Jessica Pepe, Alfredo Scillitani, Laura Tonzar, Franco Grimaldi, Renato Cozzi, Roberto Attanasio

**Affiliations:** 1 Endocrinology Unit, Azienda Sanitaria-Universitaria Friuli Centrale, P.O. Santa Maria della Misericordia, Udine, Italy;; 2 Department of Endocrinology, Diabetes and Metabolism, Ospedale Santa Croce and Carle Hospital, Cuneo, Italy;; 3 Endocrinology Department, ASST Grande Ospedale Metropolitano di Niguarda, Milan, Italy, Department of Biotechnology and Translational Medicine, University of Milan, Milan, Italy;; 4 General Oncologic and Mini-invasive Surgery Department, ASST Grande Ospedale Metropolitano di Niguarda, Milan, Italy;; 5 Department of Radiology, AOU delle Marche, Ancona, Università Politecnica delle Marche, Ancona, Italy;; 6 Department of Clinical, Internal, Anesthesiological and Cardiovascular Sciences, Sapienza University of Rome, Rome, Italy;; 7 Endocrinology Unit, Fondazione IRCCS Ca’ Granda Ospedale Maggiore Policlinico, Milan, Italy;; 8 Clinica Medica 1, Department of Medicine, University of Padova, Padova, Italy;; 9 Endocrine Surgery Unit, Department of Surgery, Oncology and Gastroenterology, University of Padova, Padova, Italy;; 10 Department of Surgical, Medical, and Molecular Pathology and Critical Care Medicine, University of Pisa, Pisa, Italy;; 11 Department of Endocrine and Ultrasound-Guided Surgery, Ospedale del Mare, Naples, Italy;; 12 Nuclear Medicine Unit, Azienda Unità Sanitaria Locale–IRCCS di Reggio Emilia, Italy;; 13 Division of Endocrinology and Diabetes Prevention and Care, IRCCS AOU di Bologna, Department of Medical and Surgical Sciences (DIMEC), Alma Mater Studiorum University of Bologna, Bologna, Italy;; 14 Department of Epidemiology, Lazio Region Health Service, Rome, Italy;; 15 High School of Economy and Management of Health Systems, Catholic University of Sacred Heart, Rome, Italy;; 16 Azienda Provinciale per i Servizi Sanitari della Provincia Autonoma di Trento, Trento, Italy;; 17 Department of Endocrinology, ULSS6 Euganea, Padova, Italy;; 18 Endocrinology, Ospedale Regina Apostolorum, Albano Laziale, Italy;; 19 Ministry of Interior - Department of Firefighters, Public Rescue and Civil Defense, Rome, Italy;; 20 Endocrinology, ASL Novara, Italy;; 21 Endocrinology, Ospedale Santo Spirito, Rome, Italy;; 22 Endocrinology and Metabolism, Agostino Gemelli University Polyclinic (IRCCS), Catholic University of the Sacred Heart, Rome, Italy;; 23 SIUEC President, Department of Surgical Sciences, University of Cagliari, Cagliari, Italy;; 24 Endocrine Unit 2, Department of Clinical and Experimental Medicine, University of Pisa, Pisa, Italy;; 25 Bone Metabolic Diseases Unit, Department of Experimental, Clinical and Biomedical Sciences, University of Florence, AOU Careggi, Florence, Italy;; 26 Bone Metabolism and Diabetes, IRCCS Istituto Auxologico Italiano, Milan, Italy, Department of Biomedical, Surgical and Dental Sciences, University of Milan, Milan, Italy;; 27 AIMN President, Nuclear Medicine Unit, AORN Ospedali dei Colli, Naples, Italy;; 28 Laboratory of Experimental Clinical Research on Bone Metabolism, Istituto Auxologico Italiano IRCCS, Milan, Italy;; 29 SIUMB President, Ultrasound Unit, S. Spirito Hospital, Pescara, Italy;; 30 General Practitioner, USL Umbria 1, Perugia, Italy;; 31 Endocrine Surgery, Ospedale Gemelli, Rome, Italy;; 32 Unit of Endocrinology, Department of Medical Specialties, Ospedale Civile di Baggiovara, Azienda Ospedaliero-Universitaria di Modena, Italy;; 33 Nephrology Unit at Policlinico Umberto I Hospital and Department of Translation and Precision Medicine, Sapienza University of Rome, Italy;; 34 Department of Emergency Radiology, Careggi University Hospital, Florence, Baggiovara, Italy;; 35 UOC Medicina Interna A, Malattie Metaboliche dell'Osso, Ambulatorio Osteoporosi e Osteopatie Fragilizzanti, Sapienza University of Rome, Rome Italy;; 36 Unit of Thyroid and Bone-Metabolic Diseases, Fondazione Policlinico Universitario Campus Bio-Medico, Rome, Italy;; 37 Unit of Endocrinology, Fondazione IRCCS Casa Sollievo della Sofferenza, San Giovanni Rotondo (FG), Italy;; 38 AME past President, Udine, Italy;; 39 AME President, Milan, Italy;; 40 AME Scientific Committee, Milan, Italy

**Keywords:** Hyperparathyroidism, sporadic, cinacalcet, bisphosphonate, surgery, parathyroidectomy, surveillance, pharmacoeconomy

## Abstract

**Aim:**

This guideline (GL) is aimed at providing a clinical practice reference for the management of sporadic primary hyperparathyroidism (PHPT) in adults. PHPT management in pregnancy was not considered.

**Methods:**

This GL has been developed following the methods described in the Manual of the Italian National Guideline System. For each question, the panel appointed by Associazione Medici Endocrinology (AME) and Società Italiana dell’Osteoporosi, del Metabolismo Minerale e delle Malattie dello Scheletro (SIOMMMS) identified potentially relevant outcomes, which were then rated for their impact on therapeutic choices. Only outcomes classified as “critical” and “important” were considered in the systematic review of evidence. Those classified as “critical” were considered for the clinical practice recommendations.

**Results:**

The present GL provides recommendations about the roles of pharmacological and surgical treatment for the clinical management of sporadic PHPT. Parathyroidectomy is recommended in comparison to surveillance or pharmacologic treatment in any adult (outside of pregnancy) or elderly subject diagnosed with sporadic PHPT who is symptomatic or meets any of the following criteria:

•  Serum calcium levels >1 mg/dL above the upper limit of normal range.
•  Urinary calcium levels >4 mg/kg/day.
•  Osteoporosis disclosed by DXA examination and/or any fragility fracture.
•  Renal function impairment (eGFR <60 mL/min).
•  Clinic or silent nephrolithiasis.
•  Age ≤50 years.

Monitoring and treatment of any comorbidity or complication of PHPT at bone, kidney, or cardiovascular level are suggested for patients who do not meet the criteria for surgery or are not operated on for any reason. Sixteen indications for good clinical practice are provided in addition to the recommendations.

**Conclusion:**

The present GL is directed to endocrinologists and surgeons - working in hospitals, territorial services or private practice - and to general practitioners and patients. The recommendations should also consider the patient’s preferences and the available resources and expertise.

## 1 INTRODUCTION

### 1.1 PHPT Definition and Epidemiology

Primary hyperparathyroidism (PHPT) is due to the autonomous hypersecretion of parathyroid hormone (PTH) with a reduction of the serum calcium inhibitory feed-back.

PHPT is the third most common endocrine disease and the most frequent cause of hypercalcemia in outpatients. PHPT is sporadic in 90-95% of cases and is originated by a single adenoma in 85% of patients and by a multiglandular disease in the remaining individuals. This guideline (GL) will not address the hereditary or genetic forms of PHPT or PHPT due to parathyroid carcinoma or atypical adenoma.

The epidemiologic profile of PHPT has changed in the last decades. Currently, the estimated incidence is 20/100,000/year, and the prevalence in the general population is 0.1-0.4%. The disease is more frequent in women, with a female-to-male ratio of 3:1, and the age peak is in the seventh decade [1, 2].

In Western countries, the clinical presentation changed in the last decades due both to the inclusion of serum calcium determination in multichannel automated assays and the increasing diffusion of the screening for osteoporosis [1, 2]. These factors resulted in the frequent finding of mild and asymptomatic conditions. Thus, presently, two main clinical presentations are diagnosed:

- Symptomatic PHPT is associated with clinically overt complications involving the target-organs of serum PTH excess, mainly the bone (skeletal fractures and brown tumors) and kidney (nephrolithiasis).
- Asymptomatic PHPT, detected by routine blood tests and no clinical evidence of disease. Asymptomatic PHPT includes complicated and uncomplicated conditions, according to the presence or absence of a clinically inapparent involvement of the target-organs.

As a further consequence of the generalized screening for osteoporosis, the condition of normocalcemic PHPT was recently described. Normocalcemic PHPT is characterized by the association of increased serum PTH levels with persistently normal serum calcium levels (anyhow measured) in the absence of any cause of secondary hyperparathyroidism. As the definition of this clinical entity is still a matter of debate, the management of normocalcemic PHPT will not be addressed in this GL [3, 4].

### 1.2 PHPT Clinical Aspects

Currently, in Italy, most PHPT patients present with asymptomatic mild hypercalcemia (<12 mg/dL), while moderate or severe hypercalcemia (12-14 mg/dL or higher than 14 mg/dL, respectively) rarely occur.

The classic targets of PTH excess are bone and kidney. While brown tumors are rarely detected, the findings of reduced bone mass, osteoporosis, and fragility fractures *-* mostly at the cortical level and asymptomatic [5, 6] *-* are frequent [7, 8]. Their prevalence is reported as high as 50-60% and is even higher in post-menopausal women [9]. Fracture risk is increased accordingly, mostly occurring at the wrist and spine [9, 10].

Nephrolithiasis occurs in 15-20% of PHPT patients [11] and is often clinically silent [12]. The main risk factor [13] for nephrolithiasis is the increase of urinary calcium excretion (>4 mg/kg/day or higher than 250 and 300 mg/day in females and males, respectively) [11], as a consequence of the enhanced PTH-mediated calcitriol production [14]. Chronic kidney disease (CKD) may be associated with PHPT in about 15% of patients [15]. CKD increases circulating PTH levels and further worsens bone mineral density (BMD) [15-17].

PHPT can also induce cardiovascular harm *-* arrhythmias, hypertension, and cardiac remodeling [9, 18-20] *-* hyperlipidemia, insulin resistance and metabolic syndrome [18, 19], neuropsychological alterations *-* depression, anxiety, irritability, brain fog, sleep disturbances, loss of concentration, dullness, psychosis, suicidal ideas, cognitive dysfunction, dementia, and hallucinations) [21-23] - and, globally, impairment of quality of life (QoL) [24, 25].

### 1.3 PHPT Diagnosis

The PHPT diagnosis is established by the association of hypercalcemia with inappropriately high levels of PTH (second or third-generation assays) both on at least two separate tests performed no less than two weeks apart. To avoid a misleading diagnosis, serum calcium should be corrected for albumin levels [26], or ionized calcium should be assayed, mostly in patients with borderline high serum calcium levels [27]. Serum phosphate levels are generally low-normal or overtly decreased due to the PTH-induced reduction of proximal renal tubular reabsorption [28].

The biochemical tests should be performed in standard conditions for the confirmation of PHPT diagnosis:

- Vitamin D repletion is defined as a serum 25-hydroxy-vitamin D level >30 ng/mL since hypovitaminosis D is a frequent cause of secondary hyperparathyroidism [29].
- Normal alimentary calcium intake since a diet with low calcium content represents a cause of secondary hyperparathyroidism [30].

A few confounding clinical conditions should also be ruled out:

- Any potentially deceiving treatment, such as the use of thiazides, lithium, and bone antiresorptive drugs [24], should be withdrawn before testing.
- Familiar hypocalciuric hypercalcemia (FHH), a genetic condition due to an inactivating mutation of the calcium-sensing receptor, should be distinguished from PHPT to avoid inappropriate surgery. The ratio between calcium clearance and creatinine clearance (ClCa/ClCr) is a clue for the differential diagnosis. A ClCa/ClCr below 0.01 should prompt a genetic analysis for FHH confirmation [31].

After a conclusive biochemical diagnosis of PHPT, imaging procedures should be employed for the localization of the affected parathyroid gland(s).

Ultrasound scan of the neck is the first-line procedure due to its low cost, wide availability, absence of radiation, and accuracy in defining the anatomical details of cervical structures. The sensitivity is 76-87%, the positive predictive value is 93-97%, and the diagnostic accuracy is up to 88% [32-34]. Limitations of cervical sonographic evaluation are the difficult visualization of specific neck regions (retro-tracheal, retro-esophageal, mediastinic) and its reduced sensitivity in patients with multinodular goiter. In addition, the technique is highly operator-dependent and requires specific expertise.

Parathyroid scintigraphy is performed either with a double phase ^99m^Tc-MIBI scan or with a double tracer (^99m^Tc-pertechnetate and ^99m^Tc-MIBI) followed by image subtraction. Single-photon emission computed tomography (SPECT) is a technical improvement that allows a tridimensional visualization of lesions.

Notably, neither ultrasound nor scintigraphy visualize the normal parathyroid glands [35].

Hybrid techniques, SPECT/computerized tomography (CT) and positron emission tomography (PET)/CT, are relevant technical progress [36-38]. The most accurate diagnostic information is provided by the ^18^F-fluorocholine PET/CT, with a 90% sensitivity and 80% detection rate, even in patients with negative or doubtful results at previous imaging, and improved safety for radioprotection [39-41]. This radiopharmaceutical is commercially available and is reimbursed by Italian National Health System (NHS).

Further second-line imaging techniques are 4D-CT [42-44] and magnetic resonance [45-47]. Both procedures can be coupled with functional imaging with ^18^F-fluorocholine PET [48]. Their role is crucial in selected clinical settings:

- Familiar PHPT (condition not addressed in this GL).
- Localization of hyperfunctioning ectopic parathyroid gland.
- Inconsistent results of first-line imaging.
- Persisting or relapsing disease after surgery.

PTH assay on the eluate from FNA (wash-out PTH) has been proposed in selected patients with ambiguous sonographic findings or who are candidates to repeat surgery after an inconclusive imaging. The procedure is not standardized and should be performed only in reference centers with specific expertise and center-specific cut-offs [49].

### 1.4 PHPT Treatment

Parathyroidectomy (PTX) is the only treatment that can achieve a PHPT cure [50]. Surgery is aimed at the resection of the pathologic parathyroid in uniglandular disease and to the subtotal or total parathyroidectomy associated with arm auto-transplantation in multiglandular disease. The primary target of PTX is the normalization of serum calcium and PTH levels.

The success rate of surgery is reported as high as 96% for uniglandular disease – but is lower for multiglandular forms – in high-volume centers (>40 PTX/year) with skilled operators [51, 52]. The surgical complication rate is low in that setting (1-3%) and includes wound infection, hemorrhage, dysphonia, and permanent hypoparathyroidism.

Bilateral neck exploration (BNE), the past standard of care, is currently substituted by surgical interventions planned on the base of pre-operative imaging findings and coupled with the use of intra-operative PTH assay (ioPTH). The rapid PTH determination demonstrates the drop of the hormone levels and confirms the correct removal of the pathologic gland. Different protocols were proposed for ioPTH, both for timing (usually at anesthesia induction and 10-15 minutes after the resection of pathologic gland) and for cut-offs. Either PTH drops higher than 50% or 70% or PTH normalization has been suggested to be predictive of PHPT cure [53]. These procedures allow a focused and safe mini-invasive approach [53]. The success rate of this technique is comparable to that of BNE but results in shorter operating times, lower rate of complications and improvement of cosmetic damage.

The recently proposed ultrasound-guided thermo-ablative techniques [54, 55] are not yet consistently standardized, are limited to a low number of centers, and their available level of evidence is still insufficient. Their use is not addressed in this GL.

The benefits of successful PTX have been largely demonstrated at bone [52, 56, 57] and renal [58, 59] levels, whereas the favorable effect of surgery is still uncertain on cardiovascular [9, 19, 21, 22, 60-62] and neuropsychological [63-69] sides. QoL was also reported as improved after surgery [70-73].

After PTX, an optimal food intake of calcium and vitamin D should be warranted. Serum calcium and PTH are generally evaluated post-operatively at day 1 to establish the need for calcium and calcitriol supply [50]. Post-operative symptomatic hypocalcemia may be due to the hungry bone syndrome and/or functional transient hypoparathyroidism [74]. In patients who are asymptomatic and with normal serum calcium levels, the level of corrected calcium should be evaluated after three and six months to confirm the cure of PHPT.

PHPT mean relapse rate after surgery is as low as <5% at 10 years [75]. The recurrence rate may be higher in young patients and in those with preoperatively unrecognized multiglandular disease [76, 77].

Disease progression is reported in the majority of PHPT patients who are followed-up without surgery. Serum calcium levels may remain stable for 10 years but eventually, an increasing trend frequently occurs [78]. BMD may similarly remain stable for up to 8 years but is reported to decrease in the long term at the femoral and radial level [78]. Finally, in elderly PHPT patients, the choice of a conservative approach is associated with an increased fracture risk [79], whereas the risk appears reduced after surgery [80]. GFR is reported to decline in mild asymptomatic PHPT patients who are not operated, with an increased risk of CKD associated with the rise of nephrolithiasis occurrence [81].

Vitamin D deficiency is more frequent in PHPT than in the general population due both to the increased activity of 1-alpha-hydroxylase – which converts calcifediol to calcitriol – and the accelerated liver metabolism of the vitamin [82]. Importantly, vitamin D deficiency is an independent risk factor for the occurrence of hungry bone syndrome after surgery [83]. In the presence of a demonstrated deficiency, vitamin D supplementation with cholecalciferol should be provided since it does not cause significant variations in serum and urinary calcium levels [84, 85].

Cinacalcet is a calcimimetic drug that potentiates the inhibitory feedback of serum calcium on parathyroid cell receptors, thus reducing serum PTH and calcium levels [86]. A systematic review of its use in PHPT patients showed serum calcium normalization in 90% of treated patients but PTH normalization only in 10% and no effect on bone or kidney outcomes [87]. Commonly reported adverse reactions after cinacalcet administration are nausea and vomiting that are mild to moderate and transient in most patients.

In PHPT patients, the treatment with alendronate, notwithstanding the lack of effect on serum or urinary calcium and PTH levels, is associated with BMD increase. However, no data are available on the occurrence of bone fractures [52, 88-91]. Denosumab, alone or in association with cinacalcet, induces a robust increase in BMD [92]. After PTX, the zoledronic acid infusion ameliorates BMD increase in comparison to the outcomes after surgery alone [93].

The efficacy of thiazides in controlling idiopathic hypercalciuria and preventing nephrolithiasis relapse [94] prompted us to consider their use in PHPT patients with hypercalciuria who are not suitable for surgery or with persisting hypercalciuria after PTX. The available data are of poor quality, and the patients treated with thiazides should be tightly controlled for serum calcium increase.

Potassium citrate supplementation was suggested for PHPT patients with nephrolithiasis, but no prospective study is available.

### 1.5 Aim of the PHPT Guideline

The aim of the present clinical practice GL is to improve patient care and support care providers by providing recommendations about the most effective and safest treatment for sporadic PHPT in adult patients.

## 2 METHODS

This GL was developed according to the Methodological manual for the production of clinical practice GLs developed by the National Center for the Clinical Excellence, Quality and Safety of Care of the Italian National Institute of Health [95, 96]. Appendix 1 lists all members of the panel, evidence review team (ERT) and external reviewers who contributed to this GL.

### 2.1 Clinical Question

The focus of recommendations is the answers to a clinical question: What is the most effective and safest treatment for sporadic PHPT in adult patients? The panel formulated the question using the Population-Intervention-Comparison-Outcome (PICO) framework (Appendix 2). We considered studies that compared surgery to no surgery or surveillance with or without pharmacotherapy.

### 2.2 Selection of Outcomes

The panel identified potentially relevant outcomes and rated the relative importance of all outcomes for decision-making using a 9-point scale. Namely:

- 1–3 points: outcomes of limited relevance
- 4–6 points: important but not critical outcomes
- 7–9 points: critical outcomes.

Only the following outcomes were classified as “critical” and considered in the systematic review of evidence and the formulation of recommendations: change in signs and symptoms of hypercalcemia, hyperparathyroidism, urolithiasis and hypercalciuria, renal damage, osteoporosis, fractures, cardio-vascular involvement, neuropsychiatric troubles, quality of life, mortality, chronic complications, permanent hypoparathyroidism, and recurrent nerve paralysis.

### 2.3 Literature Review and Assessment of the Quality of Evidence

A systematic search for each question was performed on the following databases: Cochrane Library, MEDLINE, Embase, Web of Science, and CINAHL (from inception to August 2022).

Specific search strategies were used for each database, as specified in each section of Appendix 3. No time or language limits were imposed for all the searches. References of retrieved items were searched for further studies meeting inclusion criteria.

A systematic review was performed through the following steps:

1. Selection of the eligible studies obtained with the initial search, based on title and abstract, for retrieval as full text.
2. Identification among retrieved full-text items of relevant studies, based on a priori inclusion and exclusion criteria.
3. Assessment of potential bias using validated instruments such as AMSTAR 2 for systematic reviews [97] and Cochrane tool for randomized controlled trials (RCT) [98].
4. Extraction of main characteristics of the selected studies (patient characteristics, considered outcomes, results), summarized in tables.
5. Quantitative synthesis for each outcome, calculating risk ratio (RR) for dichotomous outcomes and mean difference for continuous variables with 95% confidence intervals (CI). Data synthesis was performed with RevMan 5.4 using fixed effects models.
6. Assessment of heterogeneity by the I^2^ statistic stating the percentage of variability in effects esteem due to heterogeneity rather than to chance.
7. Assessment of the overall quality of available evidence for critical outcomes was rated using the Grading of Recommendations, Assessment, Development, and Evaluation (GRADE) approach [99]. The GRADE approach specifies four levels of the quality for a body of evidence for a given outcome:
  - High: highly reliable data whose confidence in estimated effects is unlikely to be modified by further studies.
  - Moderate: moderately reliable data whose confidence in estimated effects could be modified by further studies.
  - Low: still limited and uncertain results, which need further research for a reliable assessment of the positive and negative effects of the intervention.
  - Very low: available data are not reliable, and the estimates of effects should be considered with caution.
8. Synthesis of results was reported in the summary of findings and in the Evidence to Decision (EtD) tables, using the GRADEPro Guideline Development tool [100]. EtD tables provide a summary of the results of systematic reviews for desirable and undesirable effects of interventions, quality of available evidence, values and preferences of stakeholders, economic resources needed, equity, acceptability, and feasibility of interventions.
9. GL contents have been reported according to AGREE checklist (The Appraisal of Guidelines for REsearch & Evaluation) [101].

### 2.4 Pharmacoeconomic Studies

The economic evaluation was performed by a pharmacoeconomist with specific expertise (MB).

A survey was performed among the GL panel members from different disciplines and regions that were representative of the Italian NHS. The survey addressed the specific drivers that contribute to the total cost of each therapeutic procedure: parathyroidectomy, vitamin D, cinacalcet, bisphosphonates, denosumab, thiazides, or surveillance. Specifically, for each procedure, we investigated the duration, type and dosage of the employed drugs, type and quantities of disposable materials, number and time of involvement for each operator, and percentage of patients requiring a caregiver during and after the procedure (indirect costs).

We calculated the mean value for each parameter to allow their use in the different regional settings under NHS.

Activity-based costing (ABC) analysis was utilized to estimate the expenditures associated with the provision of the different procedures [102]. ABC consists of three steps:

1. Resource identification by means of a specific survey among interdisciplinary panelists. The resources required to implement the procedures under investigation were detailed to quantify each component (time of operators’ activities, materials, drug dosage, technical resources, *etc*.).
2. Cost measurement by consultation of scientific literature and specific databases (such as price lists).
3. Results’ valorization: the data obtained during the previous steps were combined to define the full cost of each action and of the whole process.
4. The GL economic analysis evaluated the four large resource categories employed in the procedure under investigation:
  - Direct cost paid by NHS for drugs.
  - Direct cost paid by NHS for disposable materials.
  - Direct cost paid by NHS for operators’ working time and the use of structures.
  - Indirect costs sustained by caregivers.

To assess the costs driven by the complications of treatments, we evaluated the rate of occurrence for each potential complication caused by the various procedures. The generated costs were expressed as the corresponding fraction. Namely, if the cost of a specific complication was € 5000, including all the drivers (employed drugs, hospital stay, and loss of productivity), and if the complication is reported to occur in about 1% of patients, the sum of € 50 was added to the total cost of the procedure under evaluation.

### 2.5 Development of Recommendations

Based on the evidence summarized in Etd frameworks and evidence profiles, the GL panel formulated a draft of recommendations. Any disagreements were settled through collective discussion in all cases.

The strength of recommendations is expressed as strong or weak.

A strong recommendation implies:

- For clinicians, the majority of patients should receive the recommended intervention.
- For patients: almost all properly informed patients should follow the recommendation, whereas only a small fraction of them may choose different options.
- For policy makers: the recommendation can be employed for planning the use of the available resources.

A weak recommendation implies:

- For clinicians: the final choice should include a careful consideration of patients’ values and preferences.
- For patients: the majority of properly informed patients will follow the recommendation, but a minority of them may choose different options.
- For policy makers: a discussion involving the stakeholders should be performed on the issue.

If evidence was not available or it was inappropriate for a formal grading of the quality of evidence, the GL panel developed indications for good clinical practice to be used as instructions complementary to recommendations.

### 2.6 External Review

The panel appointed an interdisciplinary board of external reviewers with specific expertise in parathyroid disease management. External reviewers received the draft version of the GL and submitted their comments to the panel, which included, after a dedicated discussion, the amendments to the GL document.

## 3 RESULTS

### 3.1 Retrieved Literature

The PRISMA flow diagram for the selection of studies is depicted in Appendix 4. Six systematic reviews were identified [52, 103-107]. The methodological quality evaluation of the selected studies is detailed in Appendix 5.

The systematic review by Ye *et al*. was selected based on relevance, updated search strategy and methodological quality [52]. It included six RCTs, whose results were reported in 12 papers [22, 65, 73, 108-116]. An updating and refining of the search strategy retrieved four news RCTs not included in the systematic review [117-120]. The RCTs compared PTX and surveillance with or without pharmacologic treatment in adult patients with mild asymptomatic or clinically overt sporadic PHPT (excluding normocalcemic PHPT).

Studies were conducted in Italy [110, 119, 120], the USA [65, 111, 112, 117], Sweden [108] and Denmark [22]. Subjects were enrolled between 1994 [112] and 2005 [110, 113] and were aged 45-80 years. Follow-up ranged between 3 months [22] and 10 years [118].

No study evaluated pharmacologic treatment as a specific comparator for surgery, but it always was administered during surveillance or while waiting for surgery.

### 3.2 Efficacy of PTX

The efficacy of PTX on serum calcium levels was evaluated in three RCTs [108, 111, 115] (213 participants; follow-up: 6 months-5 years), showing a mean reduction of 1.02 mg/dL (95%CI from -1.15 to -0.89). The efficacy of PTX on PTH levels was evaluated in the same population, showing a mean reduction of 40.54 pg/mL (95%CI from -59.35 to -21.7) after a follow-up ranging from 6 months-5 years [115] and of 41.65 pg/mL (95%CI from -62.5 to -20.79) after a follow-up ranging 6 months-10 years [50]. Serum calcium normalization was evaluated as a dichotomous result in six RCTs [108, 110-112, 115, 120] (386 participants; follow-up: 6 months-5 years), showing its achievement in 99.4% of cases. We rated the quality of this evidence as very low.

The occurrence of asymptomatic vertebral fractures was evaluated in two RCTs [110, 115] (156 participants; follow-up: 1-5 years), showing a RR of 0.18 (95%CI 0.02-1.48) and in two RCTs [110, 118] (176 participants; follow-up: 1-10 years), showing a RR of 0.89 (95%CI 0.35-2.26). The occurrence of asymptomatic non-vertebral fractures was evaluated in two RCTs [112, 115] (159 participants; follow-up: 6 months-5 years), showing an RR of 0.81 (95%CI 0.19-3.44). The occurrence of peripheral fractures was evaluated in one RCT [118] (191 participants; follow-up: 10 years) that showed a RR of 0.89 (95%CI 0.47-1.86).

The occurrence of asymptomatic renal stones was evaluated in three RCTs [110, 112, 115] (248 participants; follow-up: 1-5 years), showing an RR of 0.55 (95%CI 0.1-1.45) and in three RCTs [110, 112, 118] (294 participants) showing a RR of 0.39 (95%CI 0.11-1.45) after 10 years.

The efficacy of PTX on urinary calcium excretion was evaluated in one RCT [108] (50 participants), showing a mean reduction of 135.2 mg/24 h (95%CI from -188.69 to -81.71) after a 1-year follow-up. The efficacy of PTX on eGFR was evaluated in two RCTs [112, 118] (195 participants, showing a mean reduction of 5.52 mL/min (95%CI from -112.75 to +101.71) after 1-5 years.

QoL was evaluated in three RCTs [73, 110, 112] (225 participants), showing an improvement in all domains after 1-10 years (data not shown).

Two RCTs [109, 116] (99 participants) evaluated left ventricular mass index and ejection fraction after 1-2 years and showed a decrease of 8.04 g/m^2^ (95%CI from -17.99 to +1.91) and 2.45% (95%CI from -5.75 to +0.85), respectively. One RCT [118] (191 participants; follow-up: 10 years) evaluated cardiovascular events, arrhythmias and coronary disease, showing an RR of 1.01 (95%CI 0.44-2.32), 0.76 (95%CI 0.17-3.30), and 0.67 (95%CI 0.20-2.31), respectively.

All these results were rated with very low quality of evidence, except amelioration of serum calcium and urinary calcium excretion, and calcium normalization, which were rated as low quality of evidence.

### 3.3 Adverse Events of PTX

Only one RCT (191 participants) evaluated the risk of mortality after 5 years [115] and 10 years [118] with inconclusive evidence (RR 1.98, 95% CI 0.18-21.46, and RR 1.15, 95% CI 0.44-3.06, respectively). No study reported surgical complications, including recurrent nerve paralysis or hypoparathyroidism [108, 110, 118].

The systematic review by Ye *et al*. [52] included a review of observational studies that evaluated the occurrence of adverse events after surgery. These can be summarized as follows:

- Hematoma, 0.3%.
- Hypocalcemia is variable, ranging from 5-47%, but is generally transient.
- Long-term complications are rare: recurrent nerve injury <1% and permanent hypoparathyroidism 0-3.6%.

### 3.4 Economic Evaluation

Tables **1** and **2** show the weighted costs of surgical and non-surgical strategies for the management of PHPT, respectively.

### 3.5 Recommendations

The panel issued recommendations and indications for the management of PHPT. In clinical practice these indications should be shared with the patient after his/her complete information on the advantages and limitations of the available therapeutic options.

**Recommendation 1:** Parathyroidectomy is recommended as the best option in comparison to surveillance or pharmacologic treatment in non-pregnant adult or elderly subjects diagnosed with sporadic PHPT. Parathyroidectomy is recommended for PHPT patients who are symptomatic or who meet any of the following criteria:

- Serum calcium levels >1 mg/dL above the upper limit of normal range.
- Urinary calcium levels >4 mg/kg/day.
- Osteoporosis disclosed by DXA examination (either at the lumbar spine, femur, or ultra-distal radius level) and/or detection of any fragility fracture.
- Renal function impairment (eGFR <60 mL/min).
- Clinically overt or silent nephrolithiasis.
- Age ≤50 years.

Strong recommendation, low quality of evidence.

Monitoring and treatment of comorbidities or complications of the disease – at bone, kidney, or cardiovascular level ***-*** are suggested in patients who do not meet the recommended criteria for surgery or who are not operated on for any reason.

### 3.6 Indications for Good Clinical Practice

These statements are based on the opinion of the GL panel members about issues that are not addressed by studies directly comparing different therapeutic options. These statements are complementary to the formal recommendation, are based on large clinical experience, are unanimously agreed by the panel members, and are provided as a guide for good clinical practice.

1. PHPT diagnosis is based on the following biochemical data: increased serum calcium (either total, corrected for albumin, or ionized calcium) and PTH levels. Calcium and PTH levels should be tested after repletion of vitamin D deposits. Vitamin D deficiency may preclude the diagnosis of PHPT by inhibiting serum calcium increase above the reference range.
2. Diagnostic assessment of PHPT should be preferentially performed at a distance from acute diseases and ruling out potential drug interference.
3. Biochemical screening should be considered in first-degree relatives of young PHPT patients, due to the higher risk for a familiar form of PHPT.
4. Disease localization by imaging techniques is not required for a conclusive diagnosis of PHPT.
5. Failure of disease localization does not contraindicate surgery.
6. Fine-needle aspiration of parathyroid glands is not a routine diagnostic procedure.
7. Vitamin D repletion and adequate calcium dietary intake are advised in PHPT patients before surgery to prevent hungry bone syndrome and to improve serum PTH levels and bone turnover markers.
8. Parathyroidectomy is always appropriate in the absence of surgical contraindications, severe comorbidities or short life expectancy.
9. Parathyroidectomy should not be performed in low-volume centers that perform less than 15 parathyroid surgeries per year.
10. In patients at risk of surgical failure because of repeat surgery, negative or inconsistent preoperative localization, or suspicion of multiglandular involvement, or ectopic localization, parathyroidectomy should be performed by surgeons with specific expertise who operate in a high-volume center performing more than 40 parathyroidectomies per year.
11. Intra-operative PTH (ioPTH) assay is useful to confirm the completeness of resection during focused or unilateral surgery in patients with negative or discordant preoperative disease localization. The ioPTH assay is mandatory in patients operated upon for persisting or relapsing PHPT.
12. Clinical and biochemical follow-up can be discontinued 5 years after the resection of a single parathyroid adenoma associated with the normalization of serum albumin-corrected calcium and PTH levels. A strict and prolonged follow-up is appropriate in patients with atypical parathyroid adenomas.
13. Life-long follow-up with annual determination of albumin-corrected serum calcium and serum phosphate is appropriate in young (≤50 years) PHPT patients and in subjects with multiglandular involvement or with parathyroid hyperplasia at histology.
14. Patients who do not undergo parathyroidectomy for any reason (especially if refusing parathyroidectomy) should be informed about the risk of complications and the uncertainty of achieving a satisfactory drug control of the disease.
15. Patients who are not operated upon for any reason should receive strict life-long monitoring with:
  - Serum albumin-corrected calcium and phosphate levels and daily urinary calcium excretion at yearly intervals.
  - DXA examination and fragility fractures assessment (even asymptomatic at spine) at two-year intervals.
  - Renal function at yearly intervals.
  - Nephrolithiasis occurrence at yearly intervals. Ultrasound examination should be performed according to the initial risk and the clinical and laboratory findings and in case of symptomatic worsening.
16. The use of the following drugs should be considered:
  - Cinacalcet, when serum calcium levels are >1 mg/dL above the upper limit of normal range.
  - Bone antiresorptive drugs, according to the indications and limitations of regulatory agencies (in Italy AIFA note no.79).
  - Citrates, if calcium or uric acid stones are present.
  - Thiazides and adequate hydration, if urinary calcium excretion is increased, together with serum calcium monitoring and possible correction of sodium excretion.

### 3.7 Guideline Update

This systematic review will be updated with the use of the same search strings within three years from the date of GL approval. The ERT and the GL panel will assess the availability of new clinical data that could modify the overall quality of evidence and risk/benefit ratio and, consequently, the formulation and strength of recommendations.

The GL panel will also consider updating, adding or removing clinical questions or outcomes of interest and their relative relevance. In case of changes in clinical questions and/or critical outcomes, the process of evidence review and development of recommendations will be performed again.

## 4 DISCUSSION

Currently, PTX is the standard of care for PHPT. The mini-invasive approach is increasingly performed – primarily in case of sporadic disease – instead of traditional BNE due to the improved accuracy of surgical indication, the progress in surgical techniques, and the precision of pre-operative imaging techniques [121]. Recently, drug treatments that aim at controlling hypercalcemia and protecting bone have been made available. Thus, a non-surgical strategy can also be considered for the management of PHPT in selected patients.

The present GL analysis assessed at the best accuracy level the costs associated with the pharmacological and surgical intervention in patients affected by sporadic PHPT. A critical issue for the cost definition was the scarcity of reliable data about the best PHPT management in the Italian setting. The results of a survey addressed to clinicians with proven clinical experience within the Italian healthcare setting were used for this issue. The survey investigated the drugs, tests, visits, and the health professionals involved in the treatment process and the contribution provided by the caregiver/family members.

If PTX is the selected strategy, the estimated cost amounts to an average of € 4588. This amount includes the expense for diagnostic assessment, surgery, post-operative follow-up, and the indirect costs for the patient and the caregiver. The costs due to the acute and chronic post-operative complications that may occur in a minority of the patients – including those requiring complementary pharmacological therapies in the pre- and postoperative phase – were also covered.

In Italy, the reimbursement for the medical activities performed in public health structures is established by regulatory authorities. PTX (ICD9-CM 06.81 and 06.89) results in the maximum reimbursement of € 3482,48 for ordinary hospitalization but the costs related to long-term follow-up are not considered. By using the costs applied by the Italian NHS and taking into account the expected additional costs in the first year for the complementary services, the total expenditure can be established. The € 2356 amount calculated for the net cost of the intervention should be detracted from the € 4588 total cost for the first-year management. Then, the € 3482 sum of the NHS reimbursement should be added, so bringing the total expense induced by the surgical strategy to € 5714.

In the case of non-surgical policy, the estimated annual cost is, as a mean, € 197 and € 953 for surveillance and drug treatment, respectively.

The global costs of the different management strategies are also related to the follow-up length. As for PTX, the costs of post-surgical management should be extended for the patient’s residual life expectancy. As surgery for sporadic PHPT is performed at a mean age of 55 years [122], the residual time horizon points to about 30 years. The expenditure for the control of chronic complications may be estimated at € 10/year, and results in a total of € 300 (10 x 30) for the 30-year time horizon. Thus, the total costs for PTX and the follow-up for its complications can be estimated at € 6004 (€ 5714 + € 300 - € 10).

On the other hand, the estimated cost of the pharmacological therapy for patients who are followed up with a conservative strategy for the same 30-year time horizon amounts to € 28,590 (€ 953 x 30), while the cost of surveillance alone is estimated at € 5910 (€ 197 x 30).

It is worth noting that surgery is recommended in patients with complications. Consequently, the costs of surgical strategy should be compared primarily with those of the conservative pharmacological strategy. Thus, in PHPT the surgical approach seems more cost-effective as compared to the pharmacological treatment.

The annual incidence of PHPT is 20/100,000, so the number of new annual cases of PHPT in Italy (60 million population) may be estimated at 12,000. If the previously considered individual costs are extrapolated to the population level, in accordance with the current choice of management strategies - as indicated by the panel of experts - the annual cost of medical assistance for this disease can be estimated at over € 56 million (Table **3**).

The number of parathyroid surgeries that are performed each year in Italy is reported by the Italian Ministry of Health [123]. PTX were 2499 in 2017, 2676 in 2018, and 3115 in 2019 (before the 2020 drop to 2365, due to the COVID-19 pandemic). According to the estimates of our panel, only 3115 PHPT patients are operated upon out of the presumed 9133 candidates. Thus, every year in Italy only one-third of patients who could be candidate for surgery are actually surgically treated. Importantly, this estimate is probably in excess, because part of the parathyroid surgeries mentioned by ministerial data are not performed as a first-line surgery for sporadic PHPT. A minority of them, indeed, are addressed to PTX for recurrence or tertiary hyperparathyroidism.

Table **4** summarizes the putative net costs of the different strategies.

## CONCLUSION

The 30-year excess costs for patients who are managed pharmacologically in comparison to those who are surgically treated is therefore € 23,474 (€ 28,590 - € 5116).

If we assume that at least 10% of patients currently treated pharmacologically (n = 1494) could be switched to surgical strategy, we can estimate an annual saving of approximately € 3,497,626 (€ 23,474 x 149).

Limitations to a reliable calculation of cost estimates are:

- Price fluctuation of surgical devices and disposables.
- The risk of surgical complications and related costs, is likely higher in the real world than in the series reported by specialized centers (as defined in the indications for good clinical practice).
- Personnel costs for surgical interventions also including the pauses between operations and the non-surgical times (dressing and undressing times, patient information, informed consent, operating room cleaning, monitoring of patient weaning from anesthesia, *etc*.).
- Drop-out of the patient entering a surveillance or pharmacological strategy during their life span.
- Future price fluctuation of the drugs.
- Costs and savings of the complications that may be prevented by the surgical cure of the disease (fractures, nephrolithiasis, *etc*.).
- Indirect costs, both for the patient and the caregiver, were considered only for the surgical strategy. However, it is likely that they are similar to the other management strategies, considering the time spent on visits and controls and the hospitalizations for possible complications.

The implementation of these GL recommendations will increase the annual number of PTX, the only treatment that can achieve cure in all patients regardless of age. These changes in the therapeutic approach will improve the appropriateness of clinical actions and the efficiency in the use of the available resources for the treatment of PHPT and its complications.

## Figures and Tables

**Table 1:** Overall cost of surgical strategy for PHPT.

**Framework at Start**	Procedures for the diagnosis of PHPT	€ 561.15
	Procedures for the evaluation of comorbidities and complications	€ 326.81
	**Sub-total framework**	**€ 887.96**
**Parathyroidectomy**	Pre-surgical treatments	€ 258.51
	Procedures before hospitalization	€ 161.95
	Drugs employed during surgery	€ 44.96
	Disposables/devices	€ 206.63
	Procedures during surgery	€ 45.59
	Health professionals	€ 115.20
	Operating room	€ 193.82
	Hospital stay	€ 1,329.62
	**Sub-total surgery**	**€ 2,356.28**
**Follow-up**	Standard follow-up (95.02%)	€ 375.89
	Follow-up with acute complications (2.54%)	€ 32.79
	Follow-up with chronic complications (2.43%)	€ 10.07
	**Sub-total follow-up**	**€ 418.75**
**Indirect costs**	Patient	€ 858.21
	Care-giver	€ 66.80
	**Sub-total indirect costs**	**€ 925.01**
**Total at one year**		**€ 4,588.00**

**Table 2:** Overall annual cost of non-surgical strategy for PHPT.

**Framework at start**	Procedures for the diagnosis of PHPT	**€ 561.15**
	Procedures for the evaluation of comorbidities and complications	€ 326.81
	**Sub-total framework**	**€ 887.96**
**Non-surgical treatments**	Drugs	€ 755.92
	Follow-up	€ 197.42
	**Sub-total non-surgical treatments**	**€ 953.34**
**Total at one year**		**€ 1,841.29**

**Table 3:** Distribution of PHPT patients among the different therapeutic strategies and related costs.

**Strategy**	**%**	**N**	**Annual Cost**
Surgery	76.11	9133	€ 54,834,532
Surveillance	11.44	1373	€ 270,481
Drugs	12.45	1494	€ 1,423,782
**Total**	**100**	**12,000**	**€ 56,528,795**

**Table 4:** 30-year projection of the individual cost for PHPT patients.

**Strategy**	**Annual Cost**	**30-year Cost**
Surgery	€ 5116	€ 5116
Surveillance	€ 197	€ 5910
Drugs	€ 953	€ 28,590

## LIST OF ABBREVIATIONS

ABC: Activity Based Costing
AGREE: Appraisal of Guidelines for REsearch & Evaluation
AIMN: Associazione Italiana di Medicina Nucleare e Imaging Molecolare (Italian Association of Nuclear Imaging and Molecular Imaging)
AME: Associazione Medici Endocrinologi (Italian Association of Clinical Endocrinologists)
AMSTAR: A MeaSurement Tool to Assess systematic Reviews
BMD: Bone Mineral Density
BNE: Bilateral Neck Exploration
CI: Confidence Interval
CKD: Chronic kidney disease
CT: Computerized Tomography
DOI: Digital Object Identifier
DXA: Dual X-ray Absorptiometry
eGFR: Estimated Glomerular Filtration Rate
ERT: Evidence Review Team
EtD: Evidence to Decision
FHH: Familiar Hypocalciuric Hypercalcemia
FNA: Fine Needle Aspiration
GL: Guideline
GRADE: Grading of Recommendations Assessment, Development and Evaluation
ioPTH: Intra-Operative PTH
IRCCS: Istituto di Ricovero e Cura a Carattere Scientifico
MESH: Medical Subject Headings
NA: Not Available
NHS: National Health Service
PET: Positron Emission Tomography
PHPT: Primary Hyperparathyroidism
PICO: Population, Intervention, Comparison, Outcome
PTH: Parathyroid Hormone
PTX: Parathyroidectomy
QoL: Quality of Life
RCT: Randomized Controlled Trial
RR: Relative Risk
SF-36: Short Form-36
SIE: Società Italiana di Endocrinologia (Italian Society of Endocrinology)
SIN: Società Italiana di Nefrologia (Italian Society of Nephrology)
SIOMMMS: Società Italiana dell’Osteoporosi, del Metabolismo Minerale e delle Malattie dello Scheletro (Italian Society of Osteoporosis, Mineral Metabolism and Skeletal Diseases)
SIRM: Società Italiana di Radiologia Interventistica (Italian Society of Interventional Radiology)
SIUEC: Società Italiana Unitaria di Endocrino-Chirurgia (Italian Society of Endocrine Surgery)
SIUMB: Società Italiana di Ultrasonografia in Medicina e Biologia (Italian Society of Ultrasound in Medicine and Biology)
SPECT: Single-Photon Emission Computed Tomography

## References

[1] Walker M.D., Silverberg S.J. (2018). Primary hyperparathyroidism.. Nat. Rev. Endocrinol..

[2] Minisola S., Arnold A., Belaya Z., Brandi M.L., Clarke B.L., Hannan F.M., Hofbauer L.C., Insogna K.L., Lacroix A., Liberman U., Palermo A., Pepe J., Rizzoli R., Wermers R., Thakker R.V. (2022). Epidemiology, pathophysiology, and genetics of primary hyperparathyroidism.. J. Bone Miner. Res..

[3] Rejnmark L. (2022). European expert consensus on practical management of specific aspects of parathyroid disorders in adults and in pregnancy: Recommendations of the ESE educational program of parathyroid disorders.. Eur. J. Endocrinol..

[4] Zavatta G., Clarke B.L. (2021). Normocalcemic primary hyperparathyroidism: Need for a standardized clinical approach.. Endocrinol. Metab..

[5] Silva B.C., Costa A.G., Cusano N.E., Kousteni S., Bilezikian J.P. (2011). Catabolic and anabolic actions of parathyroid hormone on the skeleton.. J. Endocrinol. Invest..

[6] Eller-Vainicher C., Falchetti A., Gennari L., Cairoli E., Bertoldo F., Vescini F., Scillitani A., Chiodini I. (2019). DIAGNOSIS OF ENDOCRINE DISEASE: Evaluation of bone fragility in endocrine disorders.. Eur. J. Endocrinol..

[7] Cipriani C., Bilezikian J.P. (2019). Three generational phenotypes of sporadic primary hyperparathyroidism: Evolution defined by technology.. Lancet Diabetes Endocrinol..

[8] Cipriani C., Biamonte F., Costa A.G., Zhang C., Biondi P., Diacinti D., Pepe J., Piemonte S., Scillitani A., Minisola S., Bilezikian J.P. (2015). Prevalence of kidney stones and vertebral fractures in primary hyperparathyroidism using imaging technology.. J. Clin. Endocrinol. Metab..

[9] El-Hajj Fuleihan G., Chakhtoura M., Cipriani C., Eastell R., Karonova T., Liu J.M., Minisola S., Mithal A., Moreira C.A., Peacock M., Schini M., Silva B., Walker M., El Zein O., Marcocci C. (2022). Classical and nonclassical manifestations of primary hyperparathyroidism.. J. Bone Miner. Res..

[10] Narayanan N., Palui R., Merugu C., Kar S.S., Kamalanathan S., Sahoo J., Selvarajan S., Naik D. (2021). The risk of fractures in primary hyperparathyroidism: A meta-analysis.. JBMR Plus.

[11] Parks J.H., Coe F.L., Evan A.P., Worcester E.M. (2009). Clinical and laboratory characteristics of calcium stone-formers with and without primary hyperparathyroidism.. BJU Int..

[12] Cassibba S., Pellegrino M., Gianotti L., Baffoni C., Baralis E., Attanasio R., Guarnieri A., Borretta G., Tassone F. (2014). Silent renal stones in primary hyperparathyroidism: Prevalence and clinical features.. Endocr. Pract..

[13] Rejnmark L., Vestergaard P., Mosekilde L. (2011). Nephrolithiasis and renal calcifications in primary hyperparathyroidism.. J. Clin. Endocrinol. Metab..

[14] Saponaro F., Marcocci C., Apicella M., Mazoni L., Borsari S., Pardi E., Di Giulio M., Carlucci F., Scalese M., Bilezikian J.P., Cetani F. (2020). Hypomagnesuria is associated with nephrolithiasis in patients with asymptomatic primary hyperparathyroidism.. J. Clin. Endocrinol. Metab..

[15] Peacock M. (2002). Primary hyperparathyroidism and the kidney: Biochemical and clinical spectrum.. J. Bone Miner. Res..

[16] Ejlsmark-Svensson H., Bislev L.S., Rolighed L., Sikjaer T., Rejnmark L. (2018). Predictors of renal function and calcifications in primary hyperparathyroidism: A nested case-control study.. J. Clin. Endocrinol. Metab..

[17] Assadipour Y., Zhou H., Kuo E.J., Haigh P.I., Adams A.L., Yeh M.W. (2019). End-organ effects of primary hyperparathyroidism: A population-based study.. Surgery.

[18] Pepe J., Cipriani C., Sonato C., Raimo O., Biamonte F., Minisola S. (2017). Cardiovascular manifestations of primary hyperparathyroidism: A narrative review.. Eur. J. Endocrinol..

[19] Frey S., Mirallié É., Cariou B., Blanchard C. (2021). Impact of parathyroidectomy on cardiovascular risk in primary hyperparathyroidism: A narrative review.. Nutr. Metab. Cardiovasc. Dis..

[20] Verheyen N., Fahrleitner-Pammer A., Pieske B., Meinitzer A., Belyavskiy E., Wetzel J., Gaksch M., Grübler M.R., Catena C., Sechi L.A., Van Ballegooijen A.J., Brandenburg V.M., Scharnagl H., Perl S., Brussee H., März W., Pilz S., Tomaschitz A. (2016). Parathyroid hormone, aldosterone-to-renin ratio and fibroblast growth factor-23 as determinants of nocturnal blood pressure in primary hyperparathyroidism.. J. Hypertens..

[21] Sun Q., Zhang T., Chen P., Tong NW., Zhang M. (2019). Glucose metabolic disorder in primary hyperparathyroidism: A systematic review and meta-analysis.. Int. J. Clin. Exp. Med..

[22] Ejlsmark-Svensson H., Rolighed L., Rejnmark L. (2019). Effect of parathyroidectomy on cardiovascular risk factors in primary hyperparathyroidism: A randomized clinical trial.. J. Clin. Endocrinol. Metab..

[23] Cipriani C., Romagnoli E., Cilli M., Piemonte S., Pepe J., Minisola S. (2014). Quality of life in patients with primary hyperparathyroidism.. Expert Rev. Pharmacoecon. Outcomes Res..

[24] Bilezikian J.P., Silverberg S.J., Bandeira F., Cetani F., Chandran M., Cusano N.E., Ebeling P.R., Formenti A.M., Frost M., Gosnell J., Lewiecki E.M., Singer F.R., Gittoes N., Khan A.A., Marcocci C., Rejnmark L., Ye Z., Guyatt G., Potts J.T. (2022). Task Force #8. Management of primary hyperparathyroidism.. J. Bone Miner. Res..

[25] Chiodini I., Cairoli E., Palmieri S., Pepe J., Walker M.D. (2018). Non classical complications of primary hyperparathyroidism.. Best Pract. Res. Clin. Endocrinol. Metab..

[26] Bilezikian J.P., Khan A.A., Silverberg S.J., Fuleihan G.E.H., Marcocci C., Minisola S., Perrier N., Sitges-Serra A., Thakker R.V., Guyatt G., Mannstadt M., Potts J.T., Clarke B.L., Brandi M.L. (2022). Evaluation and management of primary hyperparathyroidism: Summary statement and guidelines from the fifth international workshop.. J. Bone Miner. Res..

[27] Marcocci C., Brandi M.L., Scillitani A., Corbetta S., Faggiano A., Gianotti L., Migliaccio S., Minisola S. (2015). Italian society of endocrinology consensus statement: Definition, evaluation and management of patients with mild primary hyperparathyroidism.. J. Endocrinol. Invest..

[28] Madeo B., De Vincentis S., Repaci A., Altieri P., Vicennati V., Kara E., Vescini F., Amadori P., Balestrieri A., Pagotto U., Simoni M., Rochira V. (2020). The calcium-to-phosphorous (Ca/P) ratio in the diagnosis of primary hyperparathyroidism and hypoparathyroidism: A multicentric study.. Endocrine.

[29] Zavatta G., Clarke B.L. (2020). Normocalcemic hyperparathyroidism: A heterogeneous disorder often misdiagnosed?. JBMR Plus.

[30] Shokry G., Morkos M. (2022). Calcium challenge to confirm secondary hyperparathyroidism caused by decreased calcium intake.. Endocr. Pract..

[31] Falchetti A. (2018). Genetics of parathyroids disorders: Overview.. Best Pract. Res. Clin. Endocrinol. Metab..

[32] Lee L., Steward D.L. (2010). Techniques for parathyroid localization with ultrasound.. Otolaryngol. Clin. North Am..

[33] Ha T.K., Kim D.W., Jung S.J. (2017). Ultrasound detection of normal parathyroid glands: A preliminary study.. Radiol. Med..

[34] Morris M.A., Saboury B., Ahlman M., Malayeri A.A., Jones E.C., Chen C.C., Millo C. (2022). Parathyroid imaging: Past, present, and future.. Front. Endocrinol..

[35] Gulati S., Chumber S., Puri G., Spalkit S., Damle N.A., Das C.J. (2023). Multi-modality parathyroid imaging: A shifting paradigm.. World J. Radiol..

[36] Wei W.J., Shen C.T., Song H.J., Qiu Z.L., Luo Q.Y. (2015). Comparison of SPET/CT, SPET and planar imaging using 99mTc-MIBI as independent techniques to support minimally invasive parathyroidectomy in primary hyperparathyroidism: A meta-analysis.. Hell. J. Nucl. Med..

[37] Wong K.K., Fig L.M., Gross M.D., Dwamena B.A. (2015). Parathyroid adenoma localization with 99mTc-sestamibi SPECT/CT.. Nucl. Med. Commun..

[38] Treglia G., Sadeghi R., Schalin-Jäntti C., Caldarella C., Ceriani L., Giovanella L., Eisele D.W. (2016). Detection rate of 99m Tc‐MIBI single photon emission computed tomography (SPECT)/CT in preoperative planning for patients with primary hyperparathyroidism: A meta‐analysis.. Head Neck.

[39] Beheshti M., Hehenwarter L., Paymani Z., Rendl G., Imamovic L., Rettenbacher R., Tsybrovskyy O., Langsteger W., Pirich C. (2018). 18F-Fluorocholine PET/CT in the assessment of primary hyperparathyroidism compared with 99mTc-MIBI or 99mTc-tetrofosmin SPECT/CT: A prospective dual-centre study in 100 patients.. Eur. J. Nucl. Med. Mol. Imaging.

[40] Petranović Ovčariček P., Giovanella L., Carrió Gasset I., Hindié E., Huellner M.W., Luster M., Piccardo A., Weber T., Talbot J.N., Verburg F.A. (2021). The EANM practice guidelines for parathyroid imaging.. Eur. J. Nucl. Med. Mol. Imaging.

[41] Grimaldi S., Young J., Kamenicky P., Hartl D., Terroir M., Leboulleux S., Berdelou A., Hadoux J., Hescot S., Remy H., Baudin E., Schlumberger M., Deandreis D. (2018). Challenging pre-surgical localization of hyperfunctioning parathyroid glands in primary hyperparathyroidism: The added value of 18F-Fluorocholine PET/CT.. Eur. J. Nucl. Med. Mol. Imaging.

[42] Acar N., Haciyanli M., Coskun M., Erdogan N.K., Celik S.C., Haciyanli S.G., Gur E.O. (2020). Diagnostic value of four-dimensional computed tomography and four-dimensional magnetic resonance imaging in primary hyperparathyroidism when first-line imaging was inadequate.. Ann. R. Coll. Surg. Engl..

[43] Vu T.H., Guha-Thakurta N., Harrell R.K., Ahmed S., Kumar A.J., Johnson V.E., Perrier N.D., Hamberg L.M., Hunter G.J., Schellingerhout D. (2011). Imaging characteristics of hyperfunctioning parathyroid adenomas using multiphase multidectector computed tomography: A quantitative and qualitative approach.. J. Comput. Assist. Tomogr..

[44] Kluijfhout W.P., Pasternak J.D., Beninato T., Drake F.T., Gosnell J.E., Shen W.T., Duh Q.Y., Allen I.E., Vriens M.R., de Keizer B., Hope T.A., Suh I. (2017). Diagnostic performance of computed tomography for parathyroid adenoma localization; a systematic review and meta-analysis.. Eur. J. Radiol..

[45] Nael K., Hur J., Bauer A., Khan R., Sepahdari A., Inampudi R., Guerrero M. (2015). Dynamic 4D MRI for characterization of parathyroid adenomas: Multiparametric analysis.. AJNR Am. J. Neuroradiol..

[46] Kawai Y., Iima M., Yamamoto H., Kawai M., Kishimoto A.O., Koyasu S., Yamamoto A., Omori K., Kishimoto Y. (2022). The added value of non-contrast 3-Tesla MRI for the pre-operative localization of hyperparathyroidism. Rev.. Braz J Otorhinolaryngol..

[47] Grayev A.M., Gentry L.R., Hartman M.J., Chen H., Perlman S.B., Reeder S.B. (2012). Presurgical localization of parathyroid adenomas with magnetic resonance imaging at 3.0 T: An adjunct method to supplement traditional imaging.. Ann. Surg. Oncol..

[48] Evangelista L., Ravelli I., Magnani F., Iacobone M., Giraudo C., Camozzi V., Spimpolo A., Cecchin D. (2020). 18F-choline PET/CT and PET/MRI in primary and recurrent hyperparathyroidism: A systematic review of the literature.. Ann. Nucl. Med..

[49] Castellana M., Virili C., Palermo A., Giorgino F., Giovanella L., Trimboli P. (2019). Primary hyperparathyroidism with surgical indication and negative or equivocal scintigraphy: Safety and reliability of PTH washout. A systematic review and meta-analysis.. Eur. J. Endocrinol..

[50] Perrier N., Lang B.H., Farias L.C.B., Poch L.L., Sywak M., Almquist M., Vriens M.R., Yeh M.W., Shariq O., Duh Q.Y., Yeh R., Vu T., LiVolsi V., Sitges-Serra A. (2022). Surgical aspects of primary hyperparathyroidism.. J. Bone Miner. Res..

[51] Rajan S., Gracie D., Aspinall S. (2023). Does surgeon volume impact morbidity following parathyroidectomy? A study of 16,140 parathyroidectomies from the UK Registry of Endocrine and Thyroid Surgery (UKRETS) Database.. World J. Surg..

[52] Ye Z., Silverberg S.J., Sreekanta A., Tong K., Wang Y., Chang Y., Zhang M., Guyatt G., Tangamornsuksun W., Zhang Y., Manja V., Bakaa L., Couban R.J., Brandi M.L., Clarke B., Khan A.A., Mannstadt M., Bilezikian J.P. (2022). The efficacy and safety of medical and surgical therapy in patients with primary hyperparathyroidism: A systematic review and meta-analysis of randomized controlled trials.. J. Bone Miner. Res..

[53] Rosato L., Raffaelli M., Bellantone R., Pontecorvi A., Avenia N., Boniardi M., Brandi M.L., Cetani F., Chiofalo M.G., Conzo G., De Palma M., Gasparri G., Giordano A., Innaro N., Leopaldi E., Mariani G., Marcocci C., Marini P., Miccoli P., Nasi P., Pacini F., Paragliola R., Pelizzo M.R., Testini M., De Toma G. (2014). Diagnostic, therapeutic and healthcare management protocols in parathyroid surgery: II Consensus Conference of the Italian Association of Endocrine Surgery Units (U.E.C. CLUB).. J. Endocrinol. Invest..

[54] Ye J., Huang W., Huang G., Qiu Y., Peng W., Lan N., Xie X., Liu B. (2020). Efficacy and safety of US-guided thermal ablation for primary hyperparathyroidism: A systematic review and meta-analysis.. Int. J. Hyperthermia.

[55] Chen Z., Cheng L., Zhang W., He W. (2022). Ultrasound-guided thermal ablation for hyperparathyroidism: Current status and prospects.. Int. J. Hyperthermia.

[56] Miguel G., Carranza F., Rodríguez J., Ramos M., Pablos D., Herrero E., Díaz-Guerra G. (2019). Trabecular bone score, bone mineral density and bone markers in patients with primary hyperparathyroidism 2 years after parathyroidectomy.. Horm. Metab. Res..

[57] Cusano N.E., Rubin M.R., Silva B.C., Tay Y.K.D., Williams J.M., Agarwal S., Omeragic B., Guo X.E., Bilezikian J.P. (2018). Skeletal microstructure and estimated bone strength improve following parathyroidectomy in primary hyperparathyroidism.. J. Clin. Endocrinol. Metab..

[58] Tassone F., Guarnieri A., Castellano E., Baffoni C., Attanasio R., Borretta G. (2015). Parathyroidectomy halts the deterioration of renal function in primary hyperparathyroidism.. J. Clin. Endocrinol. Metab..

[59] Matzen J., Bislev L.S., Sikjær T., Rolighed L., Hitz M.F., Eiken P., Hermann A.P., Jensen J.E.B., Abrahamsen B., Rejnmark L. (2022). The effect of parathyroidectomy compared to non-surgical surveillance on kidney function in primary hyperparathyroidism: A nationwide historic cohort study.. BMC Endocr. Disord..

[60] Pepe J., Curione M., Morelli S., Colotto M., Varrenti M., Castro C., D’Angelo A., Cipriani C., Piemonte S., Romagnoli E., Minisola S. (2013). Arrhythmias in primary hyperparathyroidism evaluated by exercise test.. Eur. J. Clin. Invest..

[61] Godang K., Lundstam K., Mollerup C., Fougner F., Pernow Y., Nordenström J., Rosén T., Jansson S., Hellström M., Bollerslev J., Heck A. (2018). The effect of surgery on fat mass, lipid and glucose metabolism in mild primary hyperparathyroidism.. Endocr. Connect..

[62] Nikooei Noghani S., Milani N., Afkhamizadeh M., Kabiri M., Bonakdaran S., Vazifeh-Mostaan L., Asadi M., Morovatdar N., Mohebbi M. (2021). Assessment of insulin resistance in patients with primary hyperparathyroidism before and after parathyroidectomy.. Endocrinol. Diabetes Metab..

[63] Roman S.A., Sosa J.A., Pietrzak R.H., Snyder P.J., Thomas D.C., Udelsman R., Mayes L. (2011). The effects of serum calcium and parathyroid hormone changes on psychological and cognitive function in patients undergoing parathyroidectomy for primary hyperparathyroidism.. Ann. Surg..

[64] Benge J.F., Perrier N.D., Massman P.J., Meyers C.A., Kayl A., Wefel J.S. (2009). Cognitive and affective sequelae of primary hyperparathyroidism and early response to parathyroidectomy.. J. Int. Neuropsychol. Soc..

[65] Perrier N.D., Balachandran D., Wefel J.S., Jimenez C., Busaidy N., Morris G.S., Dong W., Jackson E., Weaver S., Gantela S., Evans D.B., Grubbs E.G., Lee J.E. (2009). Prospective, randomized, controlled trial of parathyroidectomy versus observation in patients with “asymptomatic” primary hyperparathyroidism.. Surgery.

[66] Shah-Becker S., Derr J., Oberman B.S., Baker A., Saunders B., Carr M.M., Goldenberg D. (2018). Early neurocognitive improvements following parathyroidectomy for primary hyperparathyroidism.. Laryngoscope.

[67] Bell C.F., Warrick M.M., Gallagher K.C., Baregamian N. (2018). Neurocognitive performance profile postparathyroidectomy: A pilot study of computerized assessment.. Surgery.

[68] Liu J.Y., Saunders N.D., Chen A., Weber C.J., Sharma J. (2016). Neuropsychological changes in primary hyperparathyroidism after parathyroidectomy.. Am. Surg..

[69] Espiritu R.P., Kearns A.E., Vickers K.S., Grant C., Ryu E., Wermers R.A. (2011). Depression in primary hyperparathyroidism: Prevalence and benefit of surgery.. J. Clin. Endocrinol. Metab..

[70] Leong K.J., Sam R.C., Garnham A.W. (2010). Health-related quality of life improvement following surgical treatment of primary hyperparathyroidism in a United Kingdom population.. Surgeon.

[71] Mihai R., Sadler G.P. (2008). Pasieka’s parathyroid symptoms scores correlate with SF-36 scores in patients undergoing surgery for primary hyperparathyroidism.. World J. Surg..

[72] Pasieka J.L., Parsons L., Jones J. (2009). The long-term benefit of parathyroidectomy in primary hyperparathyroidism: A 10-year prospective surgical outcome study.. Surgery.

[73] Pretorius M., Lundstam K., Hellström M., Fagerland M.W., Godang K., Mollerup C., Fougner S.L., Pernow Y., Aas T., Hessman O., Rosén T., Nordenström J., Jansson S., Heck A., Bollerslev J. (2021). Effects of parathyroidectomy on quality of life: 10 years of data from a prospective randomized controlled trial on primary hyperparathyroidism (the SIPH-Study).. J. Bone Miner. Res..

[74] Berti P., Materazzi G., Picone A., Miccoli P. (2003). Limits and drawbacks of video-assisted parathyroidectomy.. Br. J. Surg..

[75] Hedbäck G., Odén A. (2003). Recurrence of hyperparathyroidism; a long-term follow-up after surgery for primary hyperparathyroidism.. Eur. J. Endocrinol..

[76] Lou I., Balentine C., Clarkson S., Schneider D.F., Sippel R.S., Chen H. (2017). How long should we follow patients after apparently curative parathyroidectomy?. Surgery.

[77] Van Den Heede K., Bonheure A., Brusselaers N., Van Slycke S. (2022). Long-term outcome of surgical techniques for sporadic primary hyperparathyroidism in a tertiary referral center in Belgium.. Langenbecks Arch. Surg..

[78] Rubin M.R., Bilezikian J.P., McMahon D.J., Jacobs T., Shane E., Siris E., Udesky J., Silverberg S.J. (2008). The natural history of primary hyperparathyroidism with or without parathyroid surgery after 15 years.. J. Clin. Endocrinol. Metab..

[79] Seib C.D., Meng T., Suh I., Harris A.H.S., Covinsky K.E., Shoback D.M., Trickey A.W., Kebebew E., Tamura M.K. (2022). Risk of fracture among older adults with primary hyperparathyroidism receiving parathyroidectomy vs nonoperative management.. JAMA Intern. Med..

[80] Vestergaard P., Mollerup C.L., Frøkjaer V.G., Christiansen P., Blichert-Toft M., Mosekilde L. (2000). Cohort study of risk of fracture before and after surgery for primary hyperparathyroidism.. BMJ.

[81] Yu N., Donnan P.T., Leese G.P. (2011). A record linkage study of outcomes in patients with mild primary hyperparathyroidism: The Parathyroid Epidemiology and Audit Research Study (PEARS).. Clin. Endocrinol..

[82] Clements M.R., Davies M., Fraser D.R., Lumb G.A., Mawer E.B., Adams P.H. (1987). Metabolic inactivation of vitamin D is enhanced in primary hyperparathyroidism.. Clin. Sci..

[83] Salman M.A., Rabiee A., Salman A.A., Youssef A., Shaaban H.E.D., Ftohy T., Maurice K.K., Balamoun H. (2021). Role of vitamin D supplements in prevention of hungry bone syndrome after successful parathyroidectomy for primary hyperparathyroidism: A prospective study.. Scand. J. Surg..

[84] Rolighed L., Rejnmark L., Sikjaer T., Heickendorff L., Vestergaard P., Mosekilde L., Christiansen P. (2014). Vitamin D treatment in primary hyperparathyroidism: A randomized placebo controlled trial.. J. Clin. Endocrinol. Metab..

[85] Shah V.N., Shah C.S., Bhadada S.K., Rao D.S. (2014). Effect of 25 (OH) D replacements in patients with primary hyperparathyroidism (PHPT) and coexistent vitamin D deficiency on serum 25(OH) D, calcium and PTH levels: A meta-analysis and review of literature.. Clin. Endocrinol..

[86] Hofer A.M., Brown E.M. (2003). Extracellular calcium sensing and signalling.. Nat. Rev. Mol. Cell Biol..

[87] Ng C.H., Chin Y.H., Tan M.H.Q., Ng J.X., Yang S.P., Kiew J.J., Khoo C.M. (2020). Cinacalcet and primary hyperparathyroidism: Systematic review and meta regression.. Endocr. Connect..

[88] Rossini M., Gatti D., Isaia G., Sartori L., Braga V., Adami S. (2001). Effects of oral alendronate in elderly patients with osteoporosis and mild primary hyperparathyroidism.. J. Bone Miner. Res..

[89] Chow C.C., Chan W.B., Li J.K.Y., Chan N.N., Chan M.H.M., Ko G.T.C., Lo K.W., Cockram C.S. (2003). Oral alendronate increases bone mineral density in postmenopausal women with primary hyperparathyroidism.. J. Clin. Endocrinol. Metab..

[90] Khan A.A., Bilezikian J.P., Kung A.W.C., Ahmed M.M., Dubois S.J., Ho A.Y.Y., Schussheim D., Rubin M.R., Shaikh A.M., Silverberg S.J., Standish T.I., Syed Z., Syed Z.A. (2004). Alendronate in primary hyperparathyroidism: A double-blind, randomized, placebo-controlled trial.. J. Clin. Endocrinol. Metab..

[91] Khan A.A., Bilezikian J.P., Kung A., Dubois S.J., Standish T.I., Syed Z.A. (2009). Alendronate therapy in men with primary hyperparathyroidism.. Endocr. Pract..

[92] Leere J.S., Karmisholt J., Robaczyk M., Lykkeboe S., Handberg A., Steinkohl E., Brøndum Frøkjær J., Vestergaard P. (2020). Denosumab and cinacalcet for primary hyperparathyroidism (DENOCINA): A randomised, double-blind, placebo-controlled, phase 3 trial.. Lancet Diabetes Endocrinol..

[93] Ryhänen E.M., Koski A.M., Löyttyniemi E., Välimäki M.J., Kiviniemi U., Schalin-Jäntti C. (2021). Postoperative zoledronic acid for osteoporosis in primary hyperparathyroidism: A randomized placebo-controlled study.. Eur. J. Endocrinol..

[94] Li D., Gao Y., Liu H., Huang X., Zhu R., Zhu C. (2020). Use of thiazide diuretics for the prevention of recurrent kidney calculi: A systematic review and meta-analysis.. J. Transl. Med..

[95] Excellence C. (2012). The Guidelines Manual.. http://www.ncbi.nlm.nih.gov/books/NBK395866/.

[96] Istituto Superiore di Sanità (2019). Manuale Metodologico per la produzione di linee guida di pratica clinica..

[97] Shea B.J., Reeves B.C., Wells G., Thuku M., Hamel C., Moran J., Moher D., Tugwell P., Welch V., Kristjansson E., Henry D.A. (2017). AMSTAR 2: A critical appraisal tool for systematic reviews that include randomised or non-randomised studies of healthcare interventions, or both.. BMJ.

[98] Higgins J.P.T., Altman D.G., Gøtzsche P.C., Jüni P., Moher D., Oxman A.D., Savovic J., Schulz K.F., Weeks L., Sterne J.A.C. (2011). The Cochrane Collaboration’s tool for assessing risk of bias in randomised trials.. BMJ.

[99] Guyatt G.H., Oxman A.D., Vist G.E., Kunz R., Falck-Ytter Y., Alonso-Coello P., Schünemann H.J. (2008). GRADE: An emerging consensus on rating quality of evidence and strength of recommendations.. BMJ.

[100] GRADEpro https://gradepro.org.

[101] The Appraisal of Guidelines for REsearch & Evaluation https://www.agreetrust.org/wp-content/uploads/2016/02/AGREE-Reporting-Checklist-2016.pdf.

[102] Ruggeri M., Basile M., Armuzzi A., Cicchetti A. (2016). Activity-based costing and budget analysis of vedolizumab versus conventional treatments in ulcerative colitis and Crohn’s disease.. Glob. Reg. Health Technol. Assess..

[103] Anagnostis P., Vaitsi K., Veneti S., Potoupni V., Kenanidis E., Tsiridis E., Papavramidis T.S., Goulis D.G. (2021). Efficacy of parathyroidectomy compared with active surveillance in patients with mild asymptomatic primary hyperparathyroidism: A systematic review and meta-analysis of randomized-controlled studies.. J. Endocrinol. Invest..

[104] Horiuchi K., Yoshida Y., Okamoto T. (2020). Effects of surgery on the patient-reported outcomes of primary hyperparathyroidism patients with mild hypercalcemia without classic symptoms: A systematic review of the literature.. Surg. Today.

[105] Zhang L., Liu X., Li H. (2018). Long-term skeletal outcomes of primary hyperparathyroidism patients after treatment with parathyroidectomy: A systematic review and meta-analysis.. Horm. Metab. Res..

[106] Singh Ospina N., Maraka S., Rodriguez-Gutierrez R., Espinosa de Ycaza A.E., Jasim S., Gionfriddo M., Castaneda-Guarderas A., Brito J.P., Al Nofal A., Erwin P., Wermers R., Montori V. (2016). Comparative efficacy of parathyroidectomy and active surveillance in patients with mild primary hyperparathyroidism: A systematic review and meta-analysis.. Osteoporos. Int..

[107] Cheng S.P., Lee J.J., Liu T.P., Yang P.S., Liu S.C., Hsu Y.C., Liu C.L. (2015). Quality of life after surgery or surveillance for asymptomatic primary hyperparathyroidism: A meta-analysis of randomized controlled trials.. Medicine.

[108] Almqvist E.G., Becker C., Bondeson A.G., Bondeson L., Svensson J. (2004). Early parathyroidectomy increases bone mineral density in patients with mild primary hyperparathyroidism: A prospective and randomized study.. Surgery.

[109] Almqvist E.G., Bondeson A.G., Bondeson L., Nissborg A., Smedgård P., Svensson S.E. (2002). Cardiac dysfunction in mild primary hyperparathyroidism assessed by radionuclide angiography and echocardiography before and after parathyroidectomy.. Surgery.

[110] Ambrogini E., Cetani F., Cianferotti L., Vignali E., Banti C., Viccica G., Oppo A., Miccoli P., Berti P., Bilezikian J.P., Pinchera A., Marcocci C. (2007). Surgery or surveillance for mild asymptomatic primary hyperparathyroidism: A prospective, randomized clinical trial.. J. Clin. Endocrinol. Metab..

[111] Morris G.S., Grubbs E.G., Hearon C.M., Gantela S., Lee J.E., Evans D.B., Holmes H.M., Busaidy N.L., Jimenez C., Perrier N.D. (2010). Parathyroidectomy improves functional capacity in “asymptomatic” older patients with primary hyperparathyroidism: A randomized control trial.. Ann. Surg..

[112] Rao D.S., Phillips E.R., Divine G.W., Talpos G.B. (2004). Randomized controlled clinical trial of surgery versus no surgery in patients with mild asymptomatic primary hyperparathyroidism.. J. Clin. Endocrinol. Metab..

[113] Bollerslev J., Jansson S., Mollerup C.L., Nordenström J., Lundgren E., Tørring O., Varhaug J.E., Baranowski M., Aanderud S., Franco C., Freyschuss B., Isaksen G.A., Ueland T., Rosen T. (2007). Medical observation, compared with parathyroidectomy, for asymptomatic primary hyperparathyroidism: A prospective, randomized trial.. J. Clin. Endocrinol. Metab..

[114] Lundstam K., Heck A., Godang K., Mollerup C., Baranowski M., Pernow Y., Aas T., Hessman O., Rosén T., Nordenström J., Jansson S., Hellström M., Bollerslev J. (2017). Effect of surgery versus observation: Skeletal 5-year outcomes in a randomized trial of patients with primary HPT (the SIPH Study).. J. Bone Miner. Res..

[115] Lundstam K., Heck A., Mollerup C., Godang K., Baranowski M., Pernow Y., Varhaug J.E., Hessman O., Rosén T., Nordenström J., Jansson S., Hellström M., Bollerslev J. (2015). Effects of parathyroidectomy versus observation on the development of vertebral fractures in mild primary hyperparathyroidism.. J. Clin. Endocrinol. Metab..

[116] Persson A., Bollerslev J., Rosen T., Mollerup C.L., Franco C., Isaksen G.A., Ueland T., Jansson S., Caidahl K. (2011). Effect of surgery on cardiac structure and function in mild primary hyperparathyroidism.. Clin. Endocrinol..

[117] Talpos G.B., Bone H.G., Kleerekoper M., Phillips E.R., Alam M., Honasoge M., Divine G.W., Rao D.S. (2000). Randomized trial of parathyroidectomy in mild asymptomatic primary hyperparathyroidism: Patient description and effects on the SF-36 health survey.. Surgery.

[118] Pretorius M., Lundstam K., Heck A., Fagerland M.W., Godang K., Mollerup C., Fougner S.L., Pernow Y., Aas T., Hessman O., Rosén T., Nordenström J., Jansson S., Hellström M., Bollerslev J. (2022). Mortality and morbidity in mild primary hyperparathyroidism: Results from a 10-year prospective randomized controlled trial of parathyroidectomy versus observation.. Ann. Intern. Med..

[119] Pepe J., Curione M., Morelli S., Varrenti M., Cammarota C., Cilli M., Piemonte S., Cipriani C., Savoriti C., Raimo O., De Lucia F., Colangelo L., Clementelli C., Romagnoli E., Minisola S. (2013). Parathyroidectomy eliminates arrhythmic risk in primary hyperparathyroidism, as evaluated by exercise test.. Eur. J. Endocrinol..

[120] Pepe J., Cipriani C., Curione M., Biamonte F., Colangelo L., Danese V., Cecchetti V., Sonato C., Ferrone F., Cilli M., Minisola S. (2018). Reduction of arrhythmias in primary hyperparathyroidism, by parathyroidectomy, evaluated with 24-h ECG monitoring.. Eur. J. Endocrinol..

[121] Ahmadieh H., Kreidieh O., Akl E.A., El-Hajj Fuleihan G. (2020). Minimally invasive parathyroidectomy guided by intraoperative parathyroid hormone monitoring (IOPTH) and preoperative imaging versus bilateral neck exploration for primary hyperparathyroidism in adults.. Cochrane Database Syst. Rev..

[122] Oberger M. J.V., Moreira C.A. (2020). Primary hyperparathyroidism.. Best Pract. Res. Clin. Rheumatol..

[123] Ministero della Salute La banca dati nazionale dei ricoveri ospedalieri.. https://www.salute.gov.it/portale/temi/p2_6.jsp?lingua=italiano&id=1236&area=ricoveriOspedalieri&menu=vuoto.

